# Cæsarean Operation under Difficulties

**Published:** 1896-09

**Authors:** I. P. Allred

**Affiliations:** Genoa, Fla.


### CÆSAREAN OPERATION UNDER DIFFICULTIES.

By I. P. ALLRED, M.D.,
GENOA, FLA.

On January 7, 1896, I was called to see Mrs. S. A., multipara, age twenty-eight, white. Found her under the care of an old midwife who had been with her about twenty-four hours. On examination found a case of arm presentation and dead child. The presentation had lasted four hours; the left arm and shoulder pressed against the pubic arch, head jammed in left iliac fossa, legs folded over front breech in fundus of uterus. Of course the first thing to do was to change the position so as to bring the head to the correct position. I thoroughly disinfected my hands and endeavored to return the arm, but on return of pains it would be forced back. In the intervals, while I had the arm returned, I introduced my hand into uterus and endeavored to dislodge the head from its position, but utterly failed. The force exhausted by uterus could not withstand. The arm would be forced out whenever there was a recurrence of the pains, although they were slow and weak. My next idea was resection, but taking into consideration the situation and condition of the patient, I did not believe she could live through the length of time it would take me to resect with the arm and shoulder presentation. It was now about two o'clock in the morning, and a dark, rainy, wintry night, in a log-cabin, and it was well ventilated; the wind would blow out the light every now and then, which was a small brass lamp, with no chimney. My only assistants were her husband and sister; neither could look at the operation. So the next daring idea was Cæsarean operation. Of course I recoiled with horror from this, but was I to let the woman die there for lack of courage? The patient begged me to relieve her somehow, if I had to cut it out. She had by this time become exhausted. I told them of the gravity of the operation of cutting it out, as she might die under the operation; but after laying the danger of the case before the entire family, it was decided that I go ahead and take the child from the woman as quickly as possible; it would only be death anyhow, as she was sinking fast. Pulse scarcely to be felt at wrist, so I prepared my instruments, gave her one grain morphia hypodermically and got my antiseptics ready, which were bichloride mercury and acid carbolic. My only assistants were her sister to hold back the intestines and her husband to hold the light; neither could look. The sister would hold where I put her hand. I took my scalpel and made a nine-inch incision, beginning about two inches below the navel point, down the median line just as she lay in bed; then an incision into uterus, where I found fetus driven into the inferior strait of pelvis, and so impacted that very much force had to be used to dislodge it. While I was thus engaged, with the cold January wind playing havoc with the single old brass lamplight, her sister held back the intestines with her head turned so as not to behold the awful yawning cavity that had been made in the woman's abdomen. I succeeded in emptying the uterus of its contents bruised and extravasated to an awful extent; placenta came away as the uterus proceeded to contract beautifully on the removal of fetus; it contracted to six inches in a few minutes. I next proceeded to sponge out the pelvic cavity with the antiseptic solution above mentioned, also the cavity of uterus. I then proceeded to stitch up the uterus, which took five silken stitches; returned the intestines after washing them in a tepid solution of same. Then I began at top of abdominal incision, and stitched it up with ten stitches, leaving a drainage hole at the bottom. I used no drainage-tube, as I recollected seeing somewhere if there is a rubber tube left in abdomen to come in contact with the intestines they would slough. I dressed the wound with the same antiseptic, using a piece of absorbent cotton. I had used no chloroform, ether, or any anesthesia. She stood the operation bravely, said the pain was not as great as the one she was in. She did not sink from shock. I only left Dover's powders to be given to hold back the peristaltic movement and to soothe patient, and left her resting quietly. On the 9th I saw her again, and found her resting quietly. No unusul fever; of course there was acceleration of circulatory movement. I irrigated the uterine cavity every other day for nine days, using Allen's surgical pump. Then I removed the stitches and found that I had got union by first intention most beautifully, except the drainage opening; and on washing out the uterus the solution would make its appearance through the drainage opening in abdomen; so I discontinued the irrigation and had them to use the same solution with a common female syringe twice a day. The main points about it were the small quantity of hemorrhage, no tympanitis or peritonitis, or abdominal soreness. She made a rapid and splendid recovery, with the exception of swelling of one of her legs, which was relieved without much trouble. Will say in conclusion that I kept the abdominal wound wet with the bichloride and acid carbolic solution until I removed the stitches on ninth day. Then I used iodoform and had it dressed three times per day. I never used any water from beginning to end without first being boiled. I gave her constitutional treatment from beginning.

